# The complete mitochondrial genome of *Hemerobius japonicus* (Neuroptera: Hemerobiidae)

**DOI:** 10.1080/23802359.2020.1717386

**Published:** 2020-01-29

**Authors:** Yang Zhao, Heping Shao, Ningning Zhang, Jinquan Jing

**Affiliations:** Nanjing Institute of Agricultural Sciences in Jiangsu Hilly Area, Nanjing, P.R. China

**Keywords:** Mitochondrial genome, Neuroptera, Hemerobiidae, *Hemerobius japonicus*

## Abstract

The complete mitochondrial genome of *Hemerobius japonicus* Nakahara, 1915 was sequenced in this study. The complete mitochondrial genome is a typical double-stranded circular molecule of 18,585 bp (GenBank accession number: MN852445), containing 37 typical animal mitochondrial gene and an A + T-rich region. The gene order is identical to that of the putative ancestral arrangement of insects and other lacewings. 13 protein-coding genes (PCGs) possessed common triplet initiation codons ATN except *ND1* possessed TTG and mostly terminated with TAN codons except for *ND5* and *ND4* with a single T residue adjacent to a downstream tRNA gene. All of the 22 tRNAs, ranging from 63 to 72 bp, can be folded into classic clover-leaf secondary structure except for *tRNA^Ser(AGN)^*, in which the dihydrouridine (DHU) arm did not form a stable stem-loop structure. The control region is 1416 bp long with an A + T content of 90.3%. In the sampled families of Neuroptera, each family showed a monophyletic cluster and Polystoechotidae + Rapismatidae, Osmylidae + the remaining families, Hemerobiidae + (Chrysopidae + (Polystoechotidae + Rapismatidae)) are recovered in phylogenetic analyses with high supports.

Hemerobiidae, the brown lacewings, is the third largest family of Neuroptera, with about 650 species in the world and widely distributed. The genus *Hemerobius* is the largest genus of family Hemerobiidae, with about 250 species in the world (Oswald 1993, 2019; Monserrat 2000). To date, no complete mitochondrial genome in this genus has been sequenced. In this study, we present the complete mitochondrial genome of *Hemerobius japonicus* Nakahara 1915, the first representation of genus *Hemerobius*. The samples were collected in Guyuan, Ningxia, China (35°47′22.49″N 106°17′36.99″E). Voucher specimen (No. HEME-00514) was deposited at the Entomological Lab of Nanjing Institute of Agricultural Sciences.

This mitochondrial genome is 18,585 bp long (GenBank accession number: MN852445). It includes the entire set of 37 genes (i.e. 13 protein-coding genes, 22 transfer RNA genes, and 2 ribosomal RNA genes) usually present in animal mitochondrial genomes and a control region. Gene order is identical to that of the putative ancestral arrangement of insects and other lacewings (Haruyama et al. 2011; Zhao et al. 2013, 2016; Cameron 2014). There are a total of 41 overlapped nucleotides between genes in 14 locations, ranging from 1 to 7 bp in length; while there are totally 2478 bp intergenic nucleotides in 14 locations, ranging from 1 to 2420 bp in length.

ATN, GTG, TTG, and GTT are accepted canonical mitochondrial start codons for invertebrate mtDNAs and most of the PCGs exhibit these start codons (Wolstrnholme 1992). All of the PCGs in *H. japonicus* mt genome possessed common triplet initiation codons ATN (ATG for *COII, ATP6, COIII, ND4, ND4L* and *Cytb*, ATT for *ND2, COI, ATP8, ND6*, ATA for *ND3* and *ND5*) except *ND1* possessed TTG. 11 PCGs stop with complete termination codons (9 with TAA and 2 with TAG), whereas *ND5* and *ND4* terminate with a single T residue adjacent to a downstream tRNA gene.

The *H. japonicus* mt genome contains 22 tRNA genes ranging from 63 to 72 bp. All the tRNA genes could be folded into a typical cloverleaf secondary structure except for *tRNA^Ser(AGN)^*, due to the deficiency of the dihydrouridine (DHU) arm. Among the 22 tRNA genes, 14 are encoded by the H-strand and 8 by the L-strand. The lrRNA was 1320 bp in length with an A + T content of 83.9%, while the srRNA is 781 bp long with an A + T content of 81.0%.

The control region is located between srRNA and *tRNA^Ile^* and is 1416 bp in length with an A + T content of 90.3%, which is the most AT-rich region of this mitogenome. The A + T content of the whole genome, PCGs, tRNAs, and rRNAs was 79.1%, 77.5%, 79.2%, and 82.8%, respectively.

Phylogenetic relationship was inferred from phylogenetic analysis of the 13 protein-coding genes and two rRNAs genes and generated by neighbor-joining method (NJ) of MEGA7.0. Phylogenetic analyses showed the similar relationships among sampled families as Winterton et al. (2010). Each family showed a monophyletic cluster and the following clades were highly supported (Figure 1): (1) Polystoechotidae + Rapismatidae; (2) Osmylidae + the remaining sampled families; and (3) Hemerobiidae+(Chrysopidae + (Polystoechotidae + Rapismatidae)).

**Figure 1.**
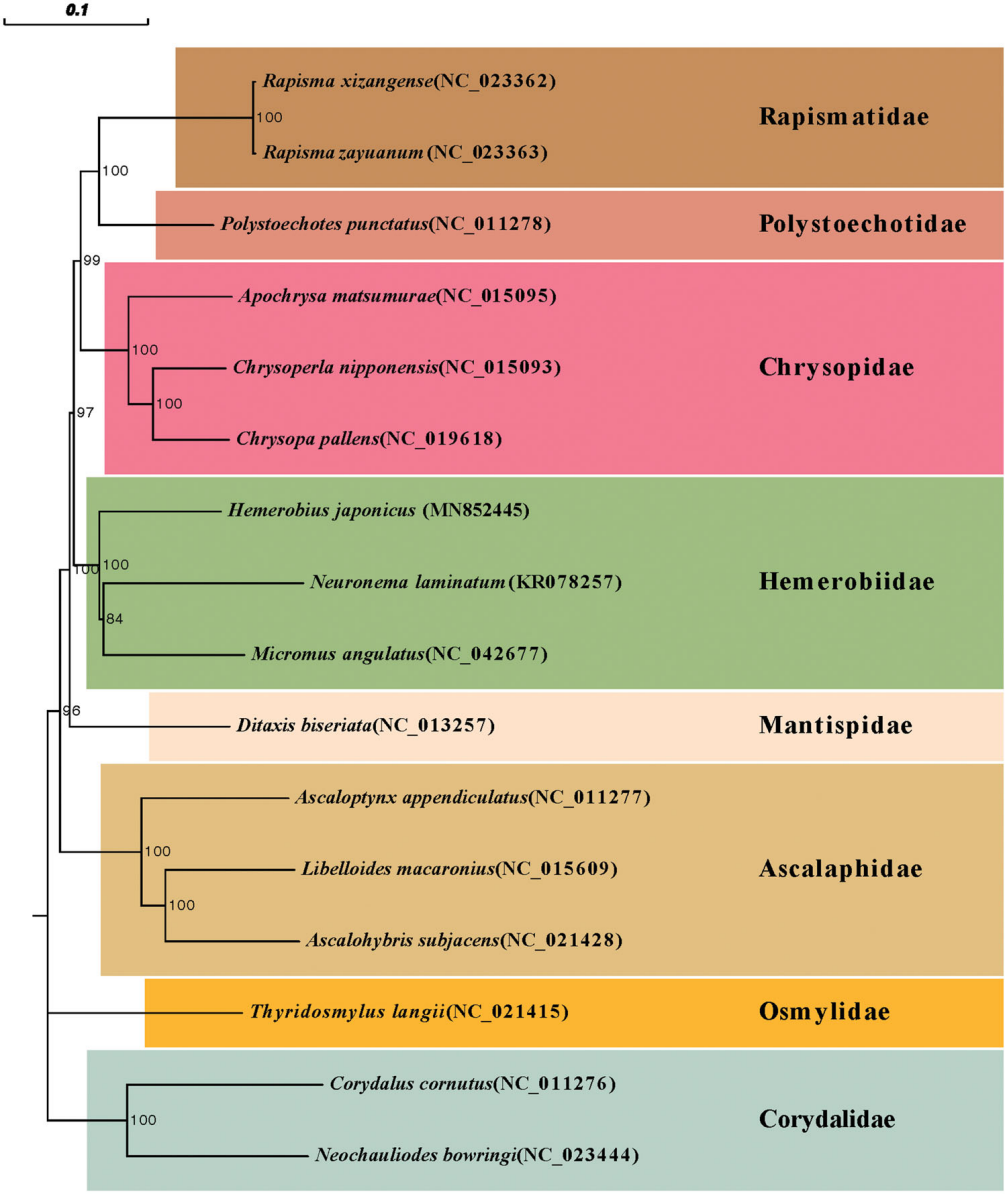
Phylogenetic relationship of seven Neuroptera families which was inferred from phylogenetic analysis of the 13 protein-coding genes and 2 rRNAs genes and generated by neighbor-joining method (NJ) of MEGA7.0. Number above each node indicates the bootstrap support values with 1000 replicates.
